# Using Behaviour Change Frameworks and Bayesian Network Modelling to Support Marine Biosecurity Practices: A New South Wales Waterways Case Study

**DOI:** 10.1007/s00267-025-02244-9

**Published:** 2025-08-07

**Authors:** J. Manyweathers, L. Hayes, G. Xie, B. Rampano, M. Hernandez-Jover

**Affiliations:** 1 School of Agricultural, Environmental and Veterinary Sciences, Charles Sturt University, Wagga Wagga, NSW Australia; 2 Gulbali Institute, Charles Sturt University, Wagga Wagga, NSW Australia; 3 Quantitative Consulting Unit, Charles Sturt University, Wagga Wagga, NSW Australia; 4 NSW Department of Primary Industries & Regional Development, Port Stephens Fisheries Institute, Anna Bay, NSW Australia

**Keywords:** Marine biosecurity, New South Wales, Recreational vessels, Bayesian Network modelling, Behaviour Change Wheel, COM-B

## Abstract

Aquatic environments are at risk of pest and disease incursions that impact livelihood and food security. Globally, recreational boats may disseminate pests and diseases from larger to smaller waterways, and their movements and biosecurity practices are less regulated than commercial boats. This project contributes to the global evidence about interventions that can support marine environmental behaviour change by capturing the practices and beliefs of owners of small to medium, permanently moored recreational boats on the New South Wales waterways to identify strategies to minimise the risk of pest and disease incursions. A three-phase mixed methods approach was adopted, beginning with stakeholder mapping and followed by key informant interviews. A Behaviour Change Wheel framework was used to develop the questionnaire tool that targeted small to medium, permanently moored recreational vessel owners. Multiple Correspondence Analysis and Bayesian Network modelling were used to segment the respondent population to enable the development of tailored communication approaches. The interview and questionnaire findings suggested that there is limited understanding by and knowledge of vessel owners about the risk of marine disease incursions. The need for clearer communication channels and stronger partnerships between stakeholders, including government agencies and industry groups, was also highlighted. Several recommendations aimed at reducing disease incursion risk were developed, including tailoring communications to segmented vessel-owning populations to address specific barriers to practice adoption. Investing in stakeholder relationships and streamlining information channels were also recommended. Future work includes the implementation and evaluation of the impact of the implemented interventions on the uptake of biosecurity practices amongst small to medium recreational vessel owners, which has ramifications for the design of interventions that support pro-environmental behaviours globally.

## Introduction

Human activity that supports biogeographic boundary movement of species is considered to play a major role in the loss of diversity, environmental change and disease incursions (Burgin and Hardiman 2011; Lewis and Maslin 2015). Aquatic environments are not excluded from these processes and are possibly more at risk due to the lack of studies examining aquatic ecosystems and the movement of pests and diseases within these environments (Ojaveer et al. 2015). Seebens et al. (2016) identified a strong correlation between the detection of pests and diseases and trade pathways, and predictions of increasing worldwide trade and transportation of goods, marine pest and disease incursions are likely to increase over the next decade (Sardain et al. 2019). Smaller, recreational vessels are likely to be involved in these incursions through secondary spread from larger commercial ports (Zabin 2014).

Biofouling is defined as the accumulation of aquatic micro-organisms, plants and animals on vessels (International Maritime Organization 2013). Biofouling on recreational vessels has the potential to transport pests and diseases through multiple water bodies, including marinas and conservation areas, and marine, fresh and brackish waters (Ferrario et al. 2017; Outinen et al. 2021). The management of biofouling on and the movement of recreational vessels are also less regular and regulated than larger, commercial, permanently moored vessels, and consequently may play a significant role in pests and disease dispersal (Clarke Murray et al. 2011; Lane et al. 2018). This is particularly significant for vessels that are permanently moored (moored after use), as opposed to vessels that are removed from the water on a trailer after use, due to the much greater potential for marine biofouling, due to the longer period spent motionless in the water.

Evidence suggests that vessel owners and operators, and marine service providers may not grasp the impact of aquatic pests and diseases (Martínez-Laiz et al. 2019). However, awareness alone is insufficient to support biofouling management behaviour change in waterway users (Golebie et al. 2021b; Payne et al. 2023). A more nuanced understanding of barriers to the management and perceptions of risk of biofouling is needed to support the design of effective interventions (Polonsky et al. 2004; Lott and Rose 2016; Golebie et al. 2023). Understanding the role of social behaviours and norms and the impact of collective action is also crucial to consider when designing interventions to support pro-environmental behaviour change (Nyborg et al. 2016; Clarke et al. 2021; Maru et al. 2025). Capturing these data to support the development of interventions that support behaviour change can be challenging, given the diverse nature of the stakeholders (Martínez-Laiz et al. 2025), but also highlights the need for considering variable approaches to reflect the different ways waterways are used and by whom (Shannon et al. 2020; Smith et al. 2023).

While there are numerous theoretical approaches that may be useful to understand the decision-making process by waterway users (Prinbeck et al. 2011), the Behaviour Change Wheel framework (Michie et al. 2014) looks at capabilities (C), opportunities (O) and motivations (M) that influence behaviour (B) (COM-B model). This model has been widely used in the environmental behaviour change arena, providing insights into barriers to and motivating factors for pro-environmental behaviour change and providing recommendations to support intervention design, including wild dog management (Hine et al. 2020) and managing urban waterways (Dorner et al. 2024; McLeod et al. 2024). The COM-B model allows for the heterogeneous nature of respondent behaviour to be better understood by considering the capability that captures the role that physical and psychological ability or self-efficacy play in whether a behaviour is adopted. Opportunity reflects how the physical and social environment might impact behaviour implementation, and motivation considers the impact of automatic (heuristics) and reflective cognitive processes on guiding behaviour (Michie et al. 2011).

The overall aim of this study was to use a behavioural framework to design and evaluate interventions that aimed to support biofouling management behaviours, using data collected from New South Wales (NSW) waterway users. Evaluation of the effectiveness and impact of the interventions was also undertaken and has been published elsewhere (Manyweathers et al. 2025). This project focused on owners/managers of small to medium-sized (up to 30 m in length), permanently moored recreational vessels.

Australia operates under a ‘Shared Responsibility’ approach to biosecurity (Rawluk et al. 2021; NSW Department of Primary industries 2020), where all citizens are expected to play a role in maintaining bioexclusion and biocontainment of pests and diseases. This is significantly different from other countries, where implementation of biosecurity practices is voluntary (Shannon et al. 2020; Vye et al. 2020). Exploring the impact of this shared responsibility approach and stakeholders’ awareness of their legal obligation (Biosecurity Duty) under the NSW *Biosecurity Act 2015* (NSW Department of Primary Industries 2020) also informed the approach to data collection, and provides a global perspective on different pathways to support biofouling management behaviours.

## Materials and Methods

A three-phase mixed methods approach was undertaken, starting with the identification and mapping of waterways stakeholders. The mapping informed the key informant interviews, which, in turn, supported the development of the cross-sectional behavioural study questionnaire targeted to owners of small to medium, permanently moored recreational vessels in the NSW waterways.

All research activities were approved by the Human Research Ethics Committee at Charles Sturt University (H20319).

### Stakeholder Identification and Mapping

Stakeholder identification and mapping were undertaken to inform a more thorough understanding of NSW waterways stakeholders, to ensure the representativeness of the subsequent work, and that the diversity of experience and opinions was captured. This activity was undertaken with the NSW Department of Primary Industries (DPI; which became the NSW Department of Primary Industries & Regional Development from 1 July 2024), Agriculture and Biosecurity Aquatic unit, and Charles Sturt University researchers, as a desktop activity. The stakeholder network was then used to inform the development of the interview tool, with the draft map validated during the key informant interviews.

### Stakeholder Interviews

The aim of the key informant interviews was to gather information on current biosecurity knowledge and practice implementation, attitudes towards biosecurity management, communication networks and values, beliefs and social norms driving these practices. Furthermore, through the interviews, information indicating the nature of the relationships between stakeholders was collected. This information was used to revise and enhance the stakeholder map and identify the strengths and direction of stakeholder relationships.

The key informant interviewees included recreational vessel owners and service providers and were recruited through NSW DPI communication networks and engagement activities. The semi-structured interview frameworks for the two stakeholder groups are presented in the Supplementary Material. The interview frameworks were reviewed by NSW DPI and piloted with two vessel owners, members of the NSW Marine Pest Working Group.

Interviews were conducted either face to face or via phone/Zoom, with interview data being uploaded to qualitative data analysis software NVivo (QSR International Pty Ltd 2018) and analysed. Qualitative data were read by two researchers, and commonalities and differences were identified.

Thematic content analysis (Boyatzis 1998; Braun et al. 2019) was then undertaken, and the outcomes from the interviews were used to inform the development of the questionnaire.

### Cross-sectional Questionnaire Development and Distribution

The target population for the cross-sectional questionnaire was small to medium-sized, permanently moored, recreational vessel owners in NSW. The questionnaire consisted of 26 questions including two demographic questions (gender, age), vessel ownership details (11 questions including size of vessel, frequency and details of use and mooring), biosecurity practices and awareness (three questions), seven questions about biosecurity actions and beliefs, including perceptions of severity of biosecurity incursions, and awareness of the impact of biofouling, and three questions about preferred and trusted information sources. The questions were designed by NSW DPI Agriculture and Biosecurity Aquatic unit, and Charles Sturt University researchers, and piloted by two different recreational vessel owners from the NSW Marine Pest Working Group, informing pre-distribution editing for clarity and validity.

Questions about possible drivers of and barriers to managing biofouling were developed (Table 1) using the COM-B model, which identifies behaviour as occurring when people have the motivation (automatic and reflective), opportunity (physical and social) and capability (psychological and physical) to undertake the behaviour, above and beyond the motivation to enact any other possible behaviours (Michie 2022).

**Table 1:** Questionnaire responses and COM-B framework categories used to build the BN model, examining barriers to undertaking biofouling activities by small to medium recreational vessel owners in NSW, 2021

	Barrier	No barrier
Capability		
Physical		
I physically cannot carry out cleaning	Agree	Neutral, Disagree
I do not have the skills	Agree	Neutral, Disagree
Psychological		
I do not know how to carry out cleaning	Agree	Neutral, Disagree
I did not know that I needed to	Agree	Neutral, Disagree
I never remember at the right time	Agree	Neutral, Disagree
I do not know who to contact	Agree	Neutral, Disagree
Opportunity		
Physical		
I do not have the time	Agree	Neutral, Disagree
It is too expensive	Agree	Neutral, Disagree
I do not have access to slip yards	Agree	Neutral, Disagree
Social		
Most people I know do not clean	Agree	Neutral, Disagree
Community expectation to clean	Disagree	Agree, Neutral
Not sure if people I know clean their vessels	Agree	Neutral, Disagree
Motivation		
Reflective		
No improvement in vessel running	Agree	Neutral, Disagree
No waterway improvement	Agree	Neutral, Disagree
Biofouling not as bad as stated	Agree	Neutral, Disagree
Cleaning ineffective	Agree	Neutral, Disagree
Waste of time	Agree	Neutral, Disagree
Automatic		
Feel no obligation to clean	Agree	Neutral, Disagree
Protecting waterways and cleaning	Disagree	Neutral, Agree
Not necessary to clean my vessel	Agree	Neutral, Agree
Right thing to do	Disagree	Neutral, Agree
Actions of good vessel owner	Disagree	Neutral, Agree

The questionnaire was distributed via invitation through NSW DPI social media and website, and shared with numerous national and state boating, sailing and marina Facebook sites, with collection taking place between December 2020 and March 2021. The questionnaire is provided in the Supplementary Material.

Data from the online survey were checked and cleaned in Excel (PC/Windows XP, 2007). Descriptive statistics were undertaken using IBM SPSS Statistics for Windows (Version 20.0. Armonk, NY: IBM Corp.) to describe characteristics and practices of participant vessel owners.

### Multiple Correspondence Analysis and Bayesian Network Model Development

Demographic variables were initially analysed using Multiple Correspondence Analysis (MCA) using R statistical software (v4.1.2; R Core Team 2021) and the resulting clusters were then used as hidden (non-observed) target variables for developing a Bayesian Network model (BN) in Netica (Norsys software Corp. 2018; R Core Team 2021). The MCA analysis involved the data reduction process of taking many variables and summarising how the variables cluster in cases over multiple axes. The analysis is based on finding the ‘centrality of the data’ (Olsen 2022, p. 98), with each variable treated as an equal component and provided with a value within the axes. Alternative positionings are tested until the optimal fit is found. This approach allows for the multidimensional nature of the data and provides researchers with the ability to distinguish between various characteristics of respondents (Olsen 2022). In this study, the MCA indicated that three clusters were optimal because they provided good differentiation of the biosecurity practices and attitudes of respondents.

Bayesian Network modelling was then used to segment the survey respondent population of small to medium-sized recreational vessel owners in NSW, and inform the development of more tailor-made strategies to support the adoption and maintenance of biosecurity practices and marine fouling management. By segmenting respondents, interventions can be targeted using a systematic, evidence-based approach (Rundle-Thiele et al. 2016; McLeod and Hine 2019), and limited resources allocated for marine disease incursion management can be used more effectively (McLeod et al. 2024).

The BN model was built using the COM-B model (Michie et al. 2011) and populated by data collected through the previously described questionnaire. Table 1 illustrates the connection between the observed variables and the specification of the hidden variables, based on identifying barriers to undertaking biofouling activities. This study utilises the most popular commercial BN software Netica (version 6.05) (Norsys Software Corp. 2018) to segment biosecurity practices and beliefs of the respondents.

The BN model was then used to explore these three clusters of vessel owners based on their self-reported capacity, motivation or opportunity to undertake biosecurity practices on their vessel, allowing a nuanced investigation of possible barriers to vessel biosecurity actions, followed by the construction of potential impactful interventions to support biosecurity practice uptake.

A BN model does not provide a solution to any lack of representativeness of data regarding the study population. It does, however, provide a mechanism to undertake exploratory analysis about marine biosecurity practices and perceptions in a consistent and systematic way. An image of the BN model used in this study is found in Supplementary Material (S1). The BN model is an interactive tool, with this figure representing the static view.

## Results

### Stakeholder Identification and Mapping

**Fig. 1:**
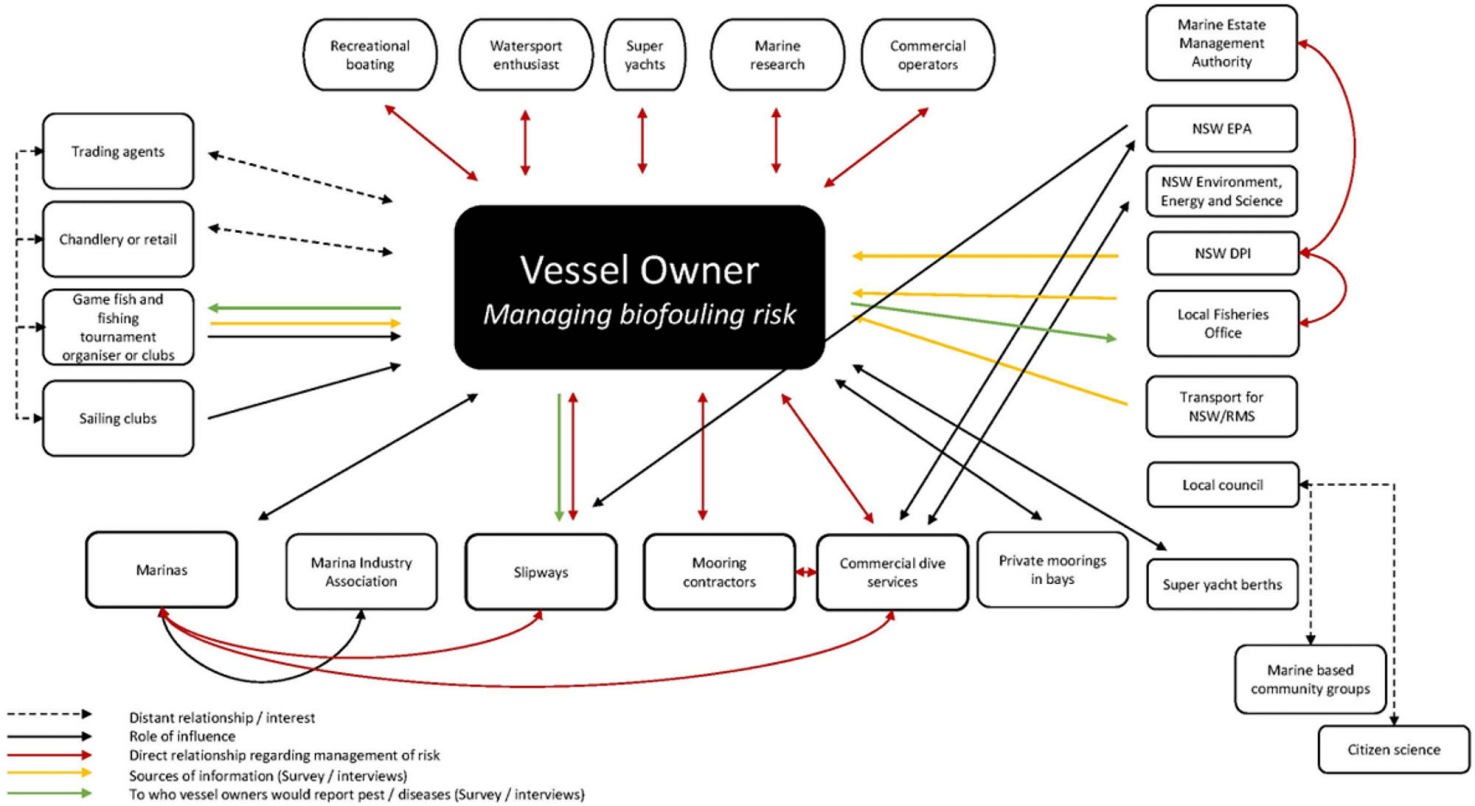
Stakeholder map representing key stakeholders and relationships in relation to small to medium-sized recreational vessel owners with permanently moored vessels in NSW and the management of biosecurity risks

The results from the stakeholder identification and mapping can be found in Fig. 1. The iterative process that developed this network highlighted the complexity of the stakeholder network and informed both the selection of interview participants and the distribution of the survey. The important role that marinas play in biosecurity management was highlighted by this process, along with the influence that game fishing clubs have within the waterways, a factor not originally identified in the draft map.

### Key Informant Interviews

The project team, using the stakeholder map as a guide, contacted 33 individuals/businesses with an invitation to participate in the interviews. Nine individuals participated, providing adequate diversity across the stakeholders, including marina owners and aquaculture operators (Table 2), with an average interview duration of 29 min.

**Table 2:** Completed interviews of NSW waterways stakeholders, 2021

Stakeholder group	Description	ID
Marina Facility Owners	Large (Clean Marina Program^a^)	S01
	Small (Clean Marina Program)	S02
	Small (non Clean Marina Program)	S03
Rec. Vessel Owners	Cruiser/Recreation/Holiday	R01 R02
Owners of other vessel types	Aquaculture operators	S04
Diving Contractor	Large	S05
Game Fishing Clubs	Far South Coast Club	S07
Fishing Charter Operators	Large NSW North Coast	S08

^a^*Clean Marina Program* = The International Clean Marina Program is an international, voluntary accreditation system for marinas, yacht clubs, boat clubs, slipways, boatyards and associated industry operators. The goal of the Clean Marina Program is to reduce ‘non-point source pollution’ associated with boating/marina facilities, and to promote clean water, clean air and thriving marina industry businesses’. https://www.marinas.net.au/accreditation-programs/what-is-the-clean-marina-program

Four main themes were developed from the interview data and include insights into vessel owners’ communication networks, attitudes towards biofouling and vessel biosecurity, barriers to and motivation for vessel cleaning, and general awareness about marine biosecurity.

#### Biosecurity Knowledge and Awareness

Participants were all aware of biosecurity and generally considered it to involve protecting their environment from pests and diseases that could be brought in. One recreational vessel owner commented that while he understood biosecurity in relation to plants and animals, he had never considered it in the context of his boating activities. There was also an understanding that their vessels could potentially spread disease from their marine environment to other areas. Those operating a commercial venture spoke of wanting to protect their livelihoods from external threats.

When asked to consider the relevance of biosecurity to everyday operations or vessel usage, responses were variable. The small Clean Marina (Table 2) operator described vessel owners as having to sign an environmental (not specifically biosecurity) policy prior to accessing a mooring, but commented that there was no protocol to check the accuracy of this. For the large marina operator, biosecurity was there but ‘in the back of your mind’ as other risks, such as the risks posed by hydrocarbons, for example, were more prominent. The fishing charter operator was very conscious of biosecurity as they travelled large distances, crossing state borders. Biosecurity was also included in the Code of Conduct of the association that this respondent represented. For the aquaculture operator, they used the term ‘blissfully ignorant’, as they had not had any previous biosecurity-related issues, as shown in the below quote.

*‘I guess we figure we’re not posing any risk to anybody else, and we don’t feel like that what we do with our, the vessels is creating any risk, we’re not bringing anything in from outside’. S04*

The recreational vessel owners differed in their responses, with one stating that the connection between biofouling management and biosecurity was highly relevant and the other admitting that:

*‘I really thought about that as a boat maintenance issue, so making sure that the boat didn’t get damaged or get sort of fouled and not be able to sail properly and that kind of thing, not about the impacts either of or from the little creepy-crawlies that are all over it’. R01*

Most participants had heard of the NSW *Biosecurity Act 2015* but were not able to clearly articulate any of its content. One respondent expressed some discontent with the number of acts and regulations that he had to comply with:

*‘There’s more Acts and regs than you can poke a stick at, but certainly I know it exists. The content of it, I don’t care to know too much about it, to be honest, because once again, what am I looking for?’ S08*

Participants were even less familiar with the general biosecurity duty, but their responses to other questions in the interview suggest that this did not necessarily mean they did not understand their responsibilities. When asked who they considered to be responsible for biosecurity in NSW waters, the dominant theme was that it was the responsibility of all who utilised the marine environment. Those interviewed assumed personal responsibility but did not have a strong sense of government responsibility for biosecurity, largely due to the number of agencies thought to play a role. One respondent went so far as to suggest that *‘…the last person I would have thought of was DPI, or Fisheries’ S08*.

There was a general awareness that there were marine pests that were notifiable, but for a number of participants, the issue was related to identification, as shown in the following quote.

*‘Yes but I don’t know what, I wouldn’t know what they look like and I wouldn’t know their names, common or, yes I wouldn’t know them off the top of my head’. S01*

#### Behaviours and Attitudes Towards Vessel Cleaning

Whilst acknowledging that there was a risk, the diving operator described a lack of enforced regulation in relation to movement and hull cleaning.

*‘…if I hopped in my boat or hopped in a boat and went to Bermagui and went to Port Stephens, at the moment there is no obligation to do anything other than just go up there and enjoy yourself. There is no obligation to hop underneath your boat and give it a rub down before you leave’. S05*

Similarly, whilst the large marina operator was aware of the importance of hull cleaning in terms of biosecurity, he indicated that any positive impact was dependent on hulls being cleaned in a controlled environment with documentation.

*‘…they need to put a layer of governance in there to say similar to your swing mooring …that you have to send through a registration - that you have to send something though to some sort of agency to say that your vessel’s been cleaned by an authorised shipyard, or – or slipway. And – and then they know that’s been controlled’. SO1*

The extent to which pest and disease transfer was something that could be controlled was questioned by the fishing charter operator.

*‘I think everything’s connected. There’s nothing in one port that won’t affect another, as far as I’m aware, so much as far as anything that fouls your vessel, like barnacles or algae growth or anything like that, because it’s – we’ve got a current that runs four knots down the east coast of Australia for six months of the year, so everything’s going to get – everything gets touched by the same water’. S08*

The small marina operator provided a service for vessel owners to have their vessels cleaned at one of three local slipways. It was noted that the costs associated with utilising the marina meant that most vessels were of high value and owners fulfilled their maintenance obligations. The large clean marina operator did not actively enforce a regime of hull cleaning; rather, they required that vessels be ‘sea worthy’.

#### Barriers and Motivating Factors Associated with Vessel Cleaning

Overwhelmingly, the greatest barriers associated with vessel cleaning were cost and access to suitable facilities. This general theme is characterised in the following statement:

*‘Well, I imagine it might be a tricky and having the infrastructure to get it out and having the manpower to be able to do it, having the equipment to be able to do it. Be a whole lot easier to go, oh, yeah, I’ll just leave it tied up’. S04*

There were concerns that a number of slipways and facilities were closing due to an inability to satisfy the NSW Environment Protection Authority (EPA) requirements.

*‘There’s not many of them around, they’ve shut so many down because of the EPA requirements, they’re not meeting these incredibly high standards Australia have in …. You wouldn’t call it a standard, it’s just ridiculous some of the things they make these people go through and people walk away from it’. S08*

Whilst this may be considered a positive in terms of protecting the marine environment from contamination, it did lead to a lack of infrastructure, which could in turn lead to cleaning being undertaken contrary to the guidelines.

Other barriers reported included the physical requirements associated with lifting and cleaning a vessel and the time required to arrange and complete the process.

*‘But yeah but how do you, how do you actually do that, making it financially viable for owners, making it time cost reasonable and time investment reasonable and keeping the fouling down, the heavy metal fouling down’. R02*

#### Communication Networks and Information Sources

When vessel owners were asked who they would contact if they had questions about an unusual marine animal or plant on their vessel, a range of options were provided, including Fisheries or Marine Parks, Google, the harbour master and NSW DPI.

In terms of experiences with receiving or accessing information on pests and diseases that pose a risk to the waterways, there was a common theme that while information may be available, it was not something that was heavily promoted by NSW DPI.

In general, participants were not certain on what to look for and considered more specific information is required in this regard.

*‘I’m sure there’s enough information out there if you go looking for it and you know what you’re looking for but for the average Joe that knows even less than I do, no’. R02*

A comparison was made by the fishing charter operator who stated that the agriculture industry was seen to provide much more visible information on the topic of pests and disease, stating that, *‘We see it everywhere else in the agricultural game’.*

The fishing group charter operator referred to commercial fishing publications that sometimes had content related to pests and disease, while also commenting that there is a gap of information considered suitable for recreational vessel owners.

*‘There’s no information for the regular Joe Blow and I’m doing it nearly every day and I don’t know a hell of a lot about it, but like what I said before, I know it exists, but the content is foreign to me’. S08*

In contrast, the aquaculture operator was satisfied, commenting:

*‘I’m quite content with the level of information about pests and so on they give us. Yeah, no, I’ve never had any reason to question the amount of information they’ve given us’. SO4*

There were mixed views on the most effective way of providing information on pests and disease, with some participants suggesting posters around marinas and others commenting that this would be unlikely to be noticed by local vessel owners.

*‘They’re just on their, they’re just doing their thing, going to their boat, they’re focused on fixing whatever they’re fixing or got to get around there to refuel or whatever it is. They’re not really keeping an eye out for new stuff. Some people might but I think it’s certainly in the minority’. R02*

One recreational vessel owner referred to the correspondence that is received in relation to registration, suggesting this as a possible pathway for distribution.

### Cross-sectional Survey Among Vessel Owners

#### Descriptive Analysis

A total of 72 questionnaire responses were received. Of these, 65 were complete enough to provide usable data. Table 3 provides a description of the demographic and general practices of survey participants. The total number of respondents to each question may vary, and some questions allowed respondents to supply more than one response.

**Table 3:** Demographic and general practices of owners of moored vessels responding to the questionnaire in the NSW waterways, 2021

	Number	Percentage
Gender	*65*	
Male	54	83.1
Female	10	15.4
Rather not say	1	1.5
Age	*64*	
18–34	4	6.3
35–50	14	21.9
51–65	31	48.4
66–80	14	21.9
Over 80	1	1.6
Length of vessel	*65*	
Less than 5 m	0	0.0
5–less than 10	25	38.5
10–less than 20	39	60.0
20–less than 30	1	1.5
30 or more	0	0.0
Mooring type	*65*	
Marina	12	18.5
Boating/yacht club	1	1.5
Private mooring	44	67.7
Private jetty	5	7.7
Commercial Mooring	2	3.1
DPI mooring	1	1.5
Source of income	*65*	
No	62	95.4
Yes hospitality	1	1.5
Yes fishing charters	1	1.5
Yes Ports/transport	1	1.5
Main use	*62*	
Cruising	30	48.4
Racing/sailing	20	32.3
Live	3	4.8
Fishing	5	8.1
Research	2	3.2
Diving/snorkelling	2	3.2
Owning duration	*65*	
<12 months	4	6.2
1–5 years	25	38.5
>5 years	36	55.4
Summer usage	*65*	
Daily	3	4.6
Once a week	39	60.0
Once a month	23	35.4
Never	0	
Winter usage	*65*	
Daily	2	3.1
Once a week	23	35.4
Once a month	39	60.0
Never	1	1.5
Mostly used	*65*	
Locally	60	92.3
Large distances across NSW	2	3.1
Interstate	1	1.5
International	2	3.1

Over 80% (*n* = 54) of participants identified as male, with the age group most represented being between 51 and 65 years of age (48.6%, *n* = 34). Most vessels owned by participants were between 10 to 20 m in length (60.0%, *n* = 39) or between 5 and 10 m in length (38.5%, *n* = 25) and were moored in private moorings (67.7%, *n* = 39) or in a marina (18.5%, *n* = 12). Over half of participants (55.4%, *n* = 36) reported owning their vessel for more than 5 years. The location of respondents, while focused in the major metropolitan regions, extended almost the full length of the NSW coastline (Supplementary Material S2).

In relation to the use of the vessels, almost all participants (95.4%, *n* = 62) did not use their vessel as a source of income, with the main use being cruising (48.4%, *n* = 30) or racing/sailing (32.3%, *n* = 20). Usage was more frequent in summer than in winter (60.0% vs. 35.4% of once a week use, respectively), and the vast majority of movements were local (92.3%, *n* = 60).

Responses to questions regarding cleaning practices are presented in Table 4. Over 80% of respondents (*n* = 52) reported removing biofouling at a slipway or on land, with most respondents choosing all that applied, indicating that they would remove biofouling at regular intervals (59.4%, *n* = 38) or when there is visible biofouling (46.9%, *n* = 30). An additional 25.0% (*n* = 16) of respondents indicated they would clean their boats when the antifouling layer paint certificate has expired. Among those cleaning at a regular interval, 60% would clean biofouling every year (*n* = 15), with 20% reporting cleaning every 2 years (*n* = 5) and another 20% reporting cleaning more than once each year (*n* = 5).

**Table 4:** Vessel cleaning-related practices of owners of a small to medium moored vessel in NSW waterways, 2021

Cleaning-related practices	Number	Percentage
Cleaning (removing biofouling) location (*non-mutually exclusive*)	*64*	
At a slipway or on land	52	81.3
In the water by a dive company	20	31.3
Never done this	2	3.1
Ever reported unusual marine pest organisms	64	
Yes	0	0.0
No	64	100.0
When would you clean biofouling from your vessel?	64	
Expiration of antifoul	16	25.0
Regular interval	38	59.4
Visible biofouling	30	46.9
Before departure	6	9.4
Before returning	4	6.3
Fuel costs increase	4	6.3
With other maintenance	10	15.6
Before selling	3	4.7
Impacts working of vessel	2	3.1
Before racing	3	4.7
Never	0	0.0
Regularity of cleaning	25	
More than once a year	5	20.0
Once a year	15	60.0
Once every 2 years	5	20.0

Respondents believed it was more likely a disease would spread from another vessel than their own vessel, indicating the perception of the risk posed by their own vessel and practices was low. However, respondents perceived the level of seriousness of a disease outbreak being the same for their own vessel or that of others. In relation to awareness of biosecurity risks, despite the vast majority reporting knowing there is a need to clean biofouling from vessels regularly (92.6%, *n* = 50), just over half indicated knowing the requirement of reporting pests and diseases (41.4%, *n* = 29) and 62.3% (*n* = 33) knew biofouling could cause damage to the health of the waterways. Less than a third of respondents were aware of the general biosecurity duty as part of the NSW *Biosecurity Act 2015* (22.9%, *n* = 16).

Responses to the questions from the COM-B framework and vessel cleaning capability indicated that most respondents disagreed that a lack of knowledge of physical skills was a barrier to undertaking biofouling management. Some respondents, however, reported limited capacity in this area, reporting that they did not have the skills to clean their vessel (22.2%, *n* = 12), did not know how to get their vessel cleaned (13.0%, *n* = 7) and did not know who to contact to get their vessel cleaned (22.2%, *n* = 12).

When asked to consider opportunities for vessel cleaning, over half of respondents agreed that there is a community expectation of cleaning biofouling from vessels (55.6%, *n* = 30), with just over a quarter being neutral (27.8%, *n* = 15). Approximately 20% of participants believed it is too expensive to clean their vessel (*n* = 11), with more than 20% indicating they were not sure if others were cleaning biofouling from their vessels (*n* = 11). In addition, 16.7% of respondents indicated their friends did not clean their vessels (*n* = 9), with 33.3% being neutral to this statement (*n* = 18). Although most respondents disagreed with the statement ‘I do not consider it necessary to clean biofouling from my vessel’ (75.9%, *n* = 41), a small proportion disagreed (9.3%, *n* = 5) or reported neutrality (14.8%, *n* = 8). These data seem to indicate that community expectations and costs are significant drivers of people’s perceptions and practices in relation to cleaning biofouling.

In general, respondents agreed that there is a need for vessel cleaning and antifouling paint, with most considering cleaning vessels as the ‘right thing to do’ and associated with being a good vessel owner (88.9%, *n* = 48; 81.5%, *n* = 44, respectively). The fact that most believed cleaning improves the vessel running costs (74.1%, *n* = 40) seems to indicate that the major motivation for cleaning vessels is financial. However, 59.3% of respondents (*n* = 32) agreed with the statement that cleaning improves waterway health, and that there is a significant risk to waterways from marine pests (44.4%, *n* = 24).

In relation to communication and information delivery practices, the three most preferred sources of information among participants were NSW DPI, the local Fisheries office and the Roads and Maritime Services (Table 5). However, only a quarter of respondents reported ever receiving information on pests and diseases from some of these sources. Information was more likely to come from the Roads and Maritime Services and NSW DPI, and participants identified these to be the most useful sources (Table 5).

**Table 5:** Communication and information delivery practices of owners of a moored boat in NSW waterways, 2021

Communication-related practice	Number	Percentage
Preferred source of information	*54*	
NSW DPI	25	47.2
Local Fisheries Office	24	45.3
RMS	21	39.6
LLS	2	3.8
NP	4	7.6
Port Authority	4	7.6
Hotline	3	5.7
Do not know	2	3.8
Google	1	1.9
Local yard/marina	3	5.7
Colleagues	2	3.8
Received information on pests and diseases?	*54*	
Yes	13	24.1
Preferred methods of information delivery	*49*	
One-on-one conversations	3	6.1
Face-2-face workshops	5	10.2
Print Newsletters	13	26.5
Electronic newsletters	37	75.5
Apps	13	26.5
Social Media	17	34.7
Websites	21	42.9

Among participants, online methods of information delivery were preferred, more specifically electronic newsletters, website, social media and apps. However, these results need to be interpreted with caution, given the survey was only distributed using online methods and may be biased towards respondents who already engage with digital/online information sources (Table 5).

### Bayesian Network Analysis

**Fig. 2:**
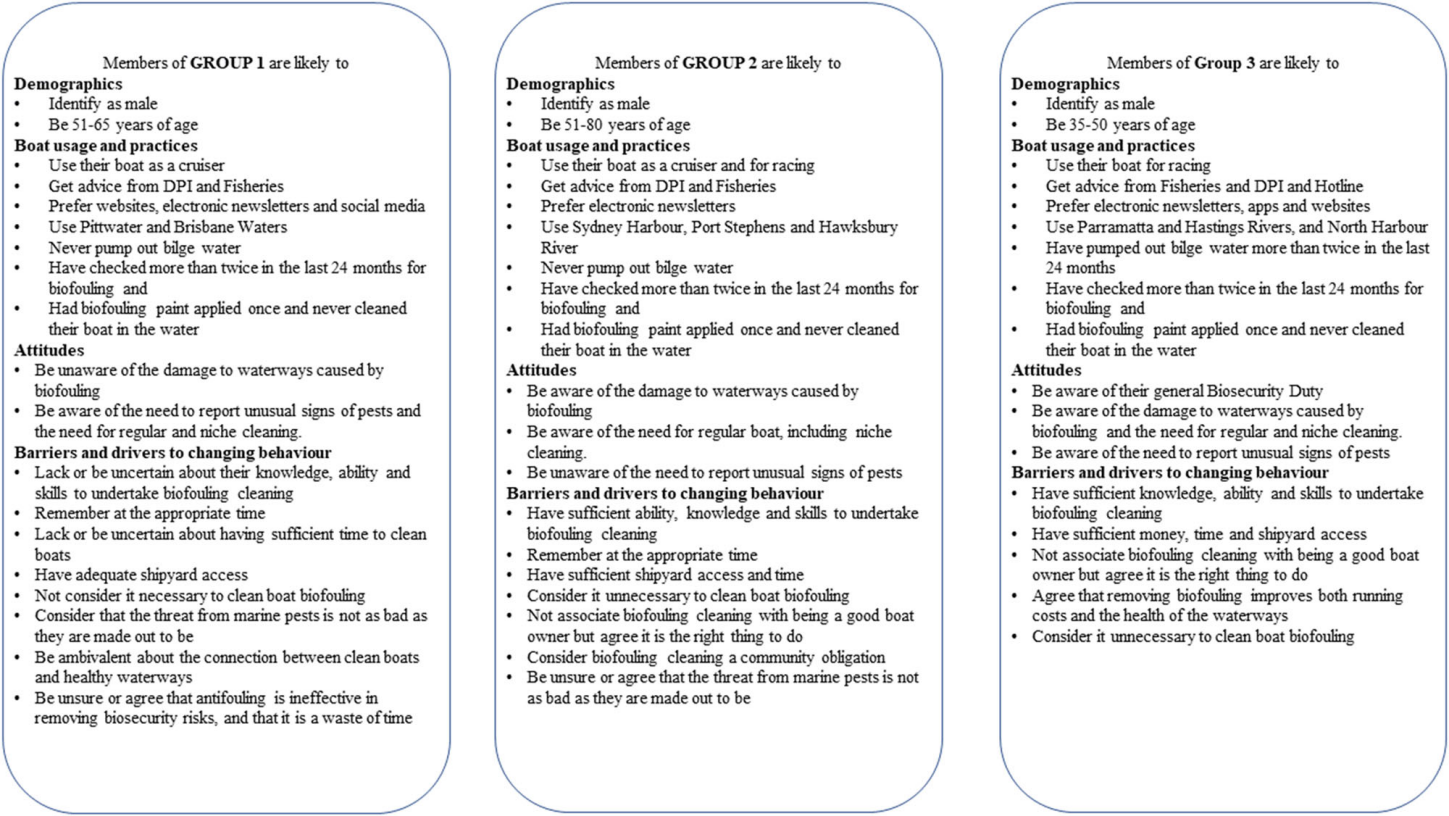
Characterisation of the current practices and potential barriers to biosecurity management behaviour change of the three demographic clusters of boat owners who responded to the biosecurity questionnaire in 2021

The BN model was used to segment the respondent population to inform the development of targeted biosecurity communication approaches. The segmented clusters are summarised in Fig. 2.

Respondents in Cluster 2 are likely to be younger but have similar vessel usage and practices as respondents in the other clusters. Cluster 2 respondents are also likely to have access, time and money for shipyard cleaning. They are also likely to be driven by a sense of community obligation to clean their vessels, but unaware of their biosecurity duty to report signs of unusual pests (NSW Department of Primary Industries 2020). Respondents from Cluster 3 were more likely to be aware of their biosecurity duty (NSW Department of Primary Industries 2020) and the connection between biofouling and damage to the waterways. They are likely to have access to shipyards and the time and financial resources to use them. Cluster 1 respondents are likely to be unsure about their capacity to undertake biofouling activities and may lack the time to do so. These respondents are also likely to be ambivalent about the effectiveness of biofouling in removing biosecurity risks, and unaware of the damage to waterways caused by biofouling.

### Sensitivity Analysis

The sensitivity of the variables to influence the determination of the respondent cluster status was estimated through the Netica’s built-in sensitivity analysis procedure. The results can be found in Table 6. The mutual information quantifies the ‘amount of information’ obtained about the target variable ‘Cluster’ by observing the other random variables. Using the sensitivity analysis, ‘Waterway’, ‘Main Use’, ‘Age’ and ‘preferring to receive communication material via electronic means’ were identified as those variables with the greatest influence on the cluster that respondents were grouped in. This means that these factors should be considered when designing communication development and distribution, with different messages and approaches adopted based on the target waterway, use of boat and information delivery preference.

**Table 6:** Bayesian Network Sensitivity Analysis of factors influencing cluster characteristics of waterway users in the New South Wales waterways, 2021

Node	Mutual information^a^	Percent
Cluster 3D	1.57334	100
Waterway	0.87567	55.7
Main use	0.35116	22.3
Age	0.34959	22.2
Pref Info delivery Electronic	0.31310	19.9
Advice from RMS^b^	0.29390	18.7
No time for cleaning	0.22781	14.5
Cleaning is a waste of time	0.21601	13.7
Mooring type	0.17865	11.4
Freq antifouling paint	0.14154	9.0
Community expectation Advice from Fisheries^c^	0.13716	8.72
	0.13683	8.7
No improvement in water	0.12340	7.84
Advice from National Parks	0.11007	7.0

^a^Mutual information (i.e. ‘entropy reduction’)—a measure of the dependence between two random variables, the changes in uncertainty of X due to knowing Y (Gómez-Villegas et al. 2014)

^b^Roads and Maritime Service—the NSW government agency historically responsible for roads and maritime infrastructure, building and traffic management

^c^Fisheries - NSW Department of Primary Industries agency responsible for NSW waterways

## Discussion

This project used a three-phase mixed methods approach to collect qualitative and quantitative data from diverse waterway stakeholders to design and evaluate behaviour change strategies to support the adoption of biofouling management practices, using NSW waterways as a case study.

The global risk to the waterways by pests and diseases has previously been identified (Lane et al. 2018; Outinen et al. 2021), together with gaps in understanding how stakeholders’ knowledge, practices and attitudes contribute to this risk (Smith et al. 2020; Golebie et al. 2021a). Understanding the intersection of sociopsychological drivers, including capability, relational, financial and cognitive factors that drive or hinder uptake of biosecurity practices by users of waterways is required to properly inform assessment and proactive and effective management of the risk of pest and disease incursions in waterways (Golebie et al. 2023; Dorner et al. 2024; McLeod et al. 2024).

The stakeholder identification and mapping exercise, interviews and questionnaire identified and confirmed several features about the case study waterway stakeholder population that are vital for understanding the biosecurity risk and informing any behaviour change process to support marine biosecurity practices, regardless of location. This project modelled how data may be collected to inform the design of such interventions and highlights the importance of evidence-informed approaches to designing interventions to ensure that they address any barriers and strengthen existing motivation.

Understanding networks and social behaviours when considering biosecurity behaviour is important (Martínez-Laiz et al. 2025; Maru et al. 2025). One significant finding from this project that demonstrates this was that game fishing clubs were found to have a higher perceived interest in small to medium recreational vessel owners and their biofouling management practices than was first identified in the mapping process, driven by their reported close connection to and desire for a healthy marine environment. These findings demonstrate the importance of gathering an in-depth, nuanced and validated understanding of networks and social behaviours when designing behaviour support interventions. In NSW, working with game fishing clubs could form a robust and previously unrecognised channel through which biosecurity information could be collected and shared.

The importance of understanding the intersection of interest and influence when considering appropriate communication channels is also demonstrated by the findings of this project (Prinbeck et al. 2011; Martínez-Laiz et al. 2025). In this study, the usual communication mechanism for marine biosecurity, NSW DPI and their local Fisheries offices, were not identified as part of the original stakeholder network mapping but were highlighted by respondents as being preferred information sources. Roads and Maritime Services also appears to have greater perceived influence on the target population than first recognised, being identified as a preferred source of information by survey respondents. A gap between those with high levels of interest in marine biosecurity (NSW DPI), including a governance and compliance role, and those identified as having a large amount of influence on boat owners, such as marinas and slipway owners/operators was also identified and the potential role for these operators needs to be reflected in future assessments of biosecurity risks and design of interventions aimed at reducing the risk posed to the natural marine environment by biofouling on recreational boats in NSW. More globally, determining where people go for their messages and who the author of the messages needs to be is important to ensure that resources to support biosecurity practices are invested wisely.

Understanding barriers that may impede the adoption of behaviour change is also important to ensure that resources and investments in such campaigns are not wasted (Golebie et al. 2023). In the current study, the interview and questionnaire data identified barriers and behavioural drivers that influence waterways users’ biosecurity practices and consequently impact the biosecurity risk. Gaps in communication networks and easy access to information on biosecurity risks and management were identified as barriers to undertaking protective behaviours. Further gaps in stakeholder knowledge about biosecurity responsibility, based on the NSW *Biosecurity Act 2015* (NSW Department of Primary Industries 2020), were also identified. This would suggest that increased investment in strengthening partnerships and connections between waterways stakeholders and NSW DPI may assist in reducing the risk of pest and disease incursion (Rawluk et al. 2021). This may result from increasing opportunities for non-compliance-related interactions between boat owners and NSW DPI, enhancing the trusted communication network that is needed for all stakeholders to report suspected pest and disease sightings and strengthen awareness of these and their impact on the waterways. While this recommendation is specific to the NSW case, any project looking to inform intervention design should consider the role of information delivery, including who the information is associated with, and the impact of that relationship on uptake of the extension material (Brewer and Ley 2012; McLeod et al. 2024).

Using the COM-B model of behaviour change is a widely used tool in biosecurity that provides connections between the barriers and motivations to certain behaviours, and interventions that may support reducing the barriers and strengthening the motivation (McLeod et al. 2024). Using this project as an example, the COM-B model, together with MCA cluster and BN analysis, can provide specific recommendations to deepen the understanding of the risk of marine pests and biofouling to waterways. For example, respondents in Cluster 1 may benefit from approaches specific to smaller boats (5 < 10 m in length) and be targeted primarily through NSW DPI e-newsletters. The content should include materials and resources that support a deeper understanding of the impact of pests and diseases on waterways and that biofouling removal is effective in supporting the waterway health. Community workshops that include stories of pests and diseases in waterways, and other concrete examples of how biofouling management can help waterways flourish, would be recommended interventions.

Cluster 2 respondents appear unlikely to be seeking information through the identified portals, which means they are either not seeking information much at all or using other avenues that are yet to be identified. Further exploration to determine the communication conduits for this cluster will be important future work. This group of boat owners can be supported through workshops and communication approaches that include examples of pests and diseases that need reporting, and approaches to reporting.

Cluster 3 respondents are likely to be younger and to have larger boats (10 < 20 m in length) and to be involved in racing. Using clubs and weekend events may be an avenue to make contact and strengthen connections with this group. They are more likely to use Roads and Maritime Services as a source of information and a diversity of preferred modes. This group is more likely to be aware of the risk of pests and diseases and what to do about them; however, their motivation to clean biofouling from their boat may be strengthened by demonstrations of the impact of clean versus dirty boats on performance.

Creation and distribution of tailored strategies that reflect an understanding of stakeholder diversity need to take place within existing hubs and trusting relationships, and evidence-based approaches to designing interventions that are built on understanding how stakeholders may perceive risk (Slovic and Peters 2016; Savadori and Lauriola 2021). This project highlighted the need for strengthening communication pathways, not just for one-way delivery of information, but to strengthen partnerships. In this study, this involved NSW DPI and Roads and Maritime Services, who were identified by participants as providing trustworthy information. More generally, the intersection of governance, compliance, social groups and risk perception needs to be considered before any interventions are designed and implemented. The role of trust in risk perception is complicated and multifactorial, with much work being undertaken on the differences between laypeople and technical experts when it comes to deciding when and how to act (Siegrist et al. 2007; Rawluk et al. 2021). Trusted relationships with technical experts in situations where participant awareness of risk is low has been found to be powerful in changing risk perception (Siegrist and Cvetkovich 2000), along with the incorporation of local knowledge into problem solving (Gates et al. 2021) and the development of such networks are recommended to enhance awareness of the biosecurity risk of biofouling.

Findings from this study indicate that while there is a developing understanding of biosecurity responsibility amongst NSW waterways stakeholders, risk perception of the impact of biofouling and knowledge of pest and disease were low. The deeper understanding of biofouling management barriers and drivers afforded by this project should be used more widely to inform the development of nuanced interventions that support positive waterway biofouling management behaviours and support preparedness for pest and disease incursions, regardless of location.

## Conclusion

This project provides a model for other waterways, both within Australia and globally, to better understand and manage biosecurity across the diversity of users and geographic locations. The current project provided a deep and nuanced look at potential barriers to strengthening practices that support marine biosecurity and reduce the risk of pest and disease incursions to NSW marine waters and recommendations to overcome some of these barriers. Recommendations include allocation of resources by NSW DPI to support positive relationship development with NSW boat owners, a diversity of communication pathways to share information about pests and diseases, and using positive imagery to encourage boat owners to manage biofouling appropriately. Implementation of these recommendations is currently underway, together with a follow-up evaluation project being undertaken in 2024 (Manyweathers et al. 2025).

## Supplementary information

NSW marine biosecurity practices_Supp mat_legends

Figure S1

Figure S2

LH01824_Marine Biosecurity interview questions

Marine survey_ Final version

## Data Availability

Due to ethics constraints and privacy, the data used in these paper cannot be made publicly available.
