# Supplementary material for: Using Behaviour Change Frameworks and Bayesian Network Modelling to Support Marine Biosecurity Practices: A New South Wales Waterways Case Study
Source: Environ Manage. 2025 Aug 7;75(12):3673–87. doi: 10.1007/s00267-025-02244-9 (PMC12575506; doi:10.1007/s00267-025-02244-9)
Supplement: Supplementary file 1 — NSW marine biosecurity practices_Supp mat_legends [file 267_2025_2244_MOESM1_ESM.docx]

**Using New South Wales marine estate stakeholder practices and attitudes to inform development of biosecurity communication and engagement activities for boat owners**

J. Manyweathers^a,b^*, L. Hayes^a^, G. Xie^c^, B. Rampano^d^ and M. Hernandez-Jover^a,b^

^a^School of Agricultural, Environmental and Veterinary Sciences, Charles Sturt University, Wagga Wagga, Australia;

^b^Gulbali Institute, Charles Sturt University, Wagga Wagga, Australia;

^c^Quantitative Consulting Unit, Charles Sturt University, Wagga Wagga, Australia;

^d^NSW Department of Primary Industries & Regional Development, Port Stephens Fisheries Institute, Australia

*corresponding author jmanyweathers@csu.edu.au

Supplementary material legends

Figure S1: A Bayesian Network model of biosecurity practices and attitude of small to medium, permanently moored recreational boat owners based in NSW in 2021

Figure S2. Location of the vessel mooring, by the nearest town and waterways, of the NSW boat owners participating in the study, 2021
