# Supplementary figures and images for: Using Behaviour Change Frameworks and Bayesian Network Modelling to Support Marine Biosecurity Practices: A New South Wales Waterways Case Study
Source: Environ Manage. 2025 Aug 7;75(12):3673–87. doi: 10.1007/s00267-025-02244-9 (PMC12575506; doi:10.1007/s00267-025-02244-9)

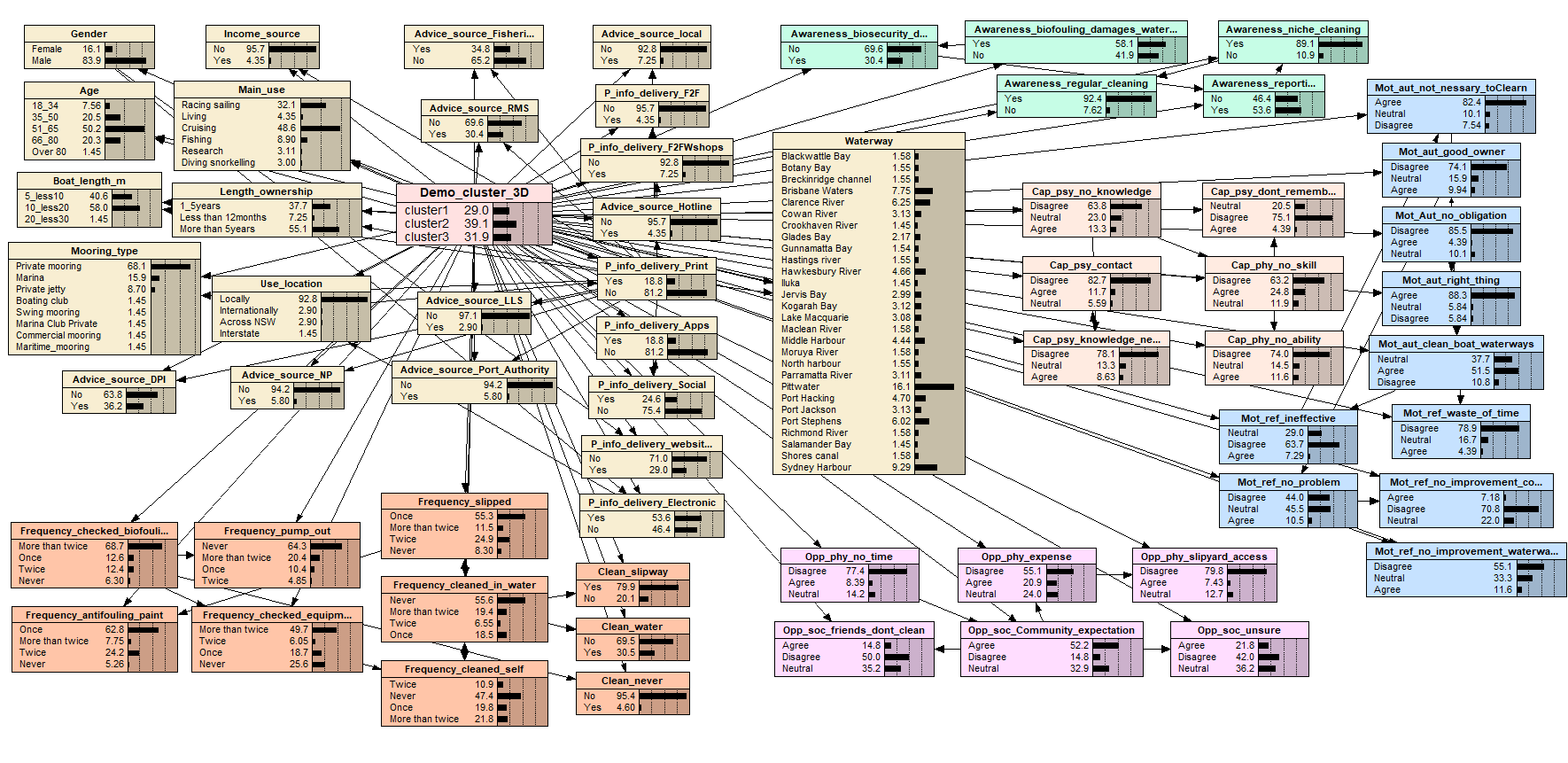

Supplement: Supplementary file 2 — Figure S1 [file 267_2025_2244_MOESM2_ESM.png]

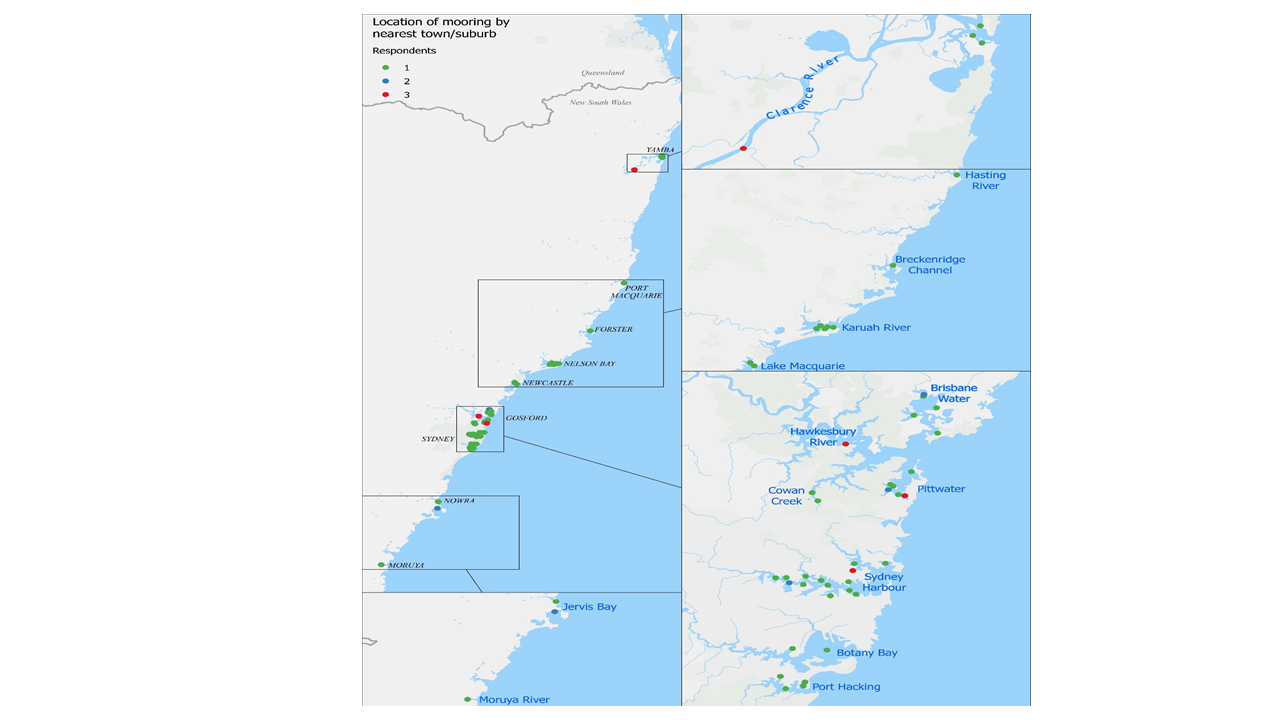

Supplement: Supplementary file 3 — Figure S2 [file 267_2025_2244_MOESM3_ESM.png]
