# Supplementary material for: Using Behaviour Change Frameworks and Bayesian Network Modelling to Support Marine Biosecurity Practices: A New South Wales Waterways Case Study
Source: Environ Manage. 2025 Aug 7;75(12):3673–87. doi: 10.1007/s00267-025-02244-9 (PMC12575506; doi:10.1007/s00267-025-02244-9)
Supplement: Supplementary file 4 — LH01824_Marine Biosecurity interview questions [file 267_2025_2244_MOESM4_ESM.pdf]

**<u>Marine Biosecurity interview questions</u>**

**Interview questions**

*(For service providers and other relevant stakeholders, excluding vessel owners)*

1. Can you please let me know about your role in your work place?
2. What do you like about your job?
3. What do you find challenging about the work you do?

*(For vessel owners)*

4. Can you please tell me why do you own a vessel? How long have you owned a vessel for? Explain us how you use your vessel (e.g. focus on movements)

*(all)*

5. Can you describe what the word "biosecurity" means for you?

*(For service providers and other relevant stakeholders, excluding vessel owners)*

6. How does biosecurity (their responses) fit into your everyday operations?

*(For vessel owners)*

7. Do you think biosecurity is relevant for how you use your vessel? OR How does biosecurity fit into your practices when you use your vessel?

*(all)*

8. Can you tell me about who you think is responsible for biosecurity of the NSW waters? (Are there other people who you think should be responsible but are not?)

9. If you think about pest and disease risk to the waterways near you, what (diseases/behaviours/attitudes/groups) concerns you the most?

10. *(If biofouling not mentioned in previous question)* Do you think biofouling poses a biosecurity risk to the NSW waters? Why? From your perspectives, how significant or common is this issue?

In thinking about cleaning vessels, including hulls/niche areas in a way that protects the waterways:

*(For vessel owners)*

11. Do you clean your vessel regularly? How and where do you clean your vessel? Do you follow any specific recommendations / guidelines for cleaning your vessel?

*(all)*

12. Do you think cleaning vessels according to guidelines makes any difference on the water ways?

13. What barriers exist that might stop you/someone from cleaning their vessel in the right way? (prompt: physical barriers – I cannot physically do; psychological barriers – I don't know how to do it, it is beyond my control, it does not make a difference, I keep forgetting)

14. What might help you/someone to be able to clean vessels? (Physical - If I was reminded, if it was easy, if I knew how, if I had easy access to a slip yard, if I had the time, if I had the money; social - if I saw others doing it, if it benefited me, no one else does it, no one I know does it)

15. What might motivate you/someone to clean vessels? (Autonomic - not my responsibility, it is the right thing to do, if I saw a benefit to it; Reflective – I love the ocean, I know it is the law, I do not want to get punished by Fisheries, I always mean to do it)

16. If you had questions about an unusual marine animal/plant on your boat, who would you go to for information and advice? Why? Who wouldn't you go to? Why?

17. Have you ever received information on pests and diseases posing a risk to the waterways? If yes, from who? Was the information useful? Do you think there is enough information available on the risks and how to manage them? What are the gaps?

18. Did you know that there are invasive marine organisms/animal/plant and that these should be reported to NSW DPI?

19. Have you ever reported an unusual marine organisms/animal/plant to anyone? If yes, who did you report it to?

20. Are you aware of the NSW Biosecurity Act 2015? If yes, are you aware of what the General Biosecurity Duty is and how this apply to all of us?

21. Are you part of any biosecurity program or activity either professionally or personally? (Eg, citizen science project, Clean Marinas etc.)

22. Is there anything else in relation to pest / animal biosecurity risks and practices that you would like to discuss that we have not covered in the previous questions?
