# Supplementary material for: Using Behaviour Change Frameworks and Bayesian Network Modelling to Support Marine Biosecurity Practices: A New South Wales Waterways Case Study
Source: Environ Manage. 2025 Aug 7;75(12):3673–87. doi: 10.1007/s00267-025-02244-9 (PMC12575506; doi:10.1007/s00267-025-02244-9)
Supplement: Supplementary file 5 — Marine survey_ Final version [file 267_2025_2244_MOESM5_ESM.pdf]

Marine Biosecurity Awareness – Social research study

Before we get started, we just need to make sure that this survey is right for you.

Do you own (or manage on behalf of an owner) a boat that STAYS moored or at a berth in coastal NSW waters for any period of time.

- ◯ Yes
- ◯ No

Marine Biosecurity Awareness – Social research study

The following information describes the who, what and why of the research. Please use the Previous and Next buttons at the bottom of each page to navigate through the survey.

Principal Investigator
Associate Professor Marta Hernandez-Jover BVSc, MSc, PhD. Associate Professor Veterinary Epidemiology & Public Health, School of Animal and Veterinary Sciences, Charles Sturt University. Graham Centre for Agricultural Innovation
E: mhernandez-jover@csu.edu.au P: 02 6933 2086

Co-investigators
-Ben Rampano Aquatic Biosecurity Policy & Projects Officer. NSW Department of Primary Industries, Port Stephens Fisheries Institute
-Melissa Walker Team Leader, Aquatic Policy & Programs. NSW Department of Primary Industries, Port Stephens Fisheries Institute
-Karina Worrell Aquatic Biosecurity Policy & Projects Officer. NSW Department of Primary Industries, Port Stephens Fisheries Institute
-Gayle Garbutt Aquatic Biosecurity Policy & Projects Officer NSW Department of Primary Industries, Port Stephens Fisheries Institute
-Dr Jennifer Manyweathers School of Animal and Veterinary Sciences, Charles Sturt University and Graham Centre for Agricultural Innovation
-Lynne Hayes School of Animal and Veterinary Sciences, Charles Sturt University and Graham Centre for Agricultural Innovation

You are invited to participate in a research study looking at marine biosecurity awareness. Please note that to participate in the research you must be over 18 years old.The study is being conducted by the researchers listed at the top of this document from Charles Sturt University, the Graham Centre for Agricultural Innovation

and NSW Department of Primary Industries.

Before you decide whether or not you wish to participate, it is important for you to understand why the research is being done and what it will involve. Please take the time to read the following information carefully and discuss it with others if you wish.

What is the purpose of this study?
This study aims to gather information on what people think about the management of marine fouling organisms on small-medium sized vessels and the risk of spread of marine pest and diseases in the marine estate across NSW. This information will then to be used to prepare recommendations for a campaign to reduce the risk of spread of marine pest and diseases.

Why have I been invited to participate in this study?
We are inviting participation from owners of small-medium sized vessels (we are not including those operating large commercial vessels, international shipping and recreational trailer boat vessels)

What does this study involve?
The study involves completing an online survey. We expect that the survey will take approximately 20 minutes to complete.

Are there any risks and benefits to me in taking part in this study?
Participation in this study should involve no physical or mental discomfort. However, if as a result of participating in this research you feel you need help or to talk to someone, we suggest that you contact your GP or counsellor. Beyond Blue ca

How will my confidentiality be protected?

The survey will be completed anonymously with no identifying information collected. All information collected from you will be stored securely and only accessed by the researchers unless you consent otherwise. Data will be retained for at least 5 years after final publication at Charles Sturt University, the Graham Centre for Agricultural Innovation and NSW Department of Primary Industries.

How is this study being paid for?

This social research was funded by the NSW Government under the Marine Estate Management Strategy. The ten-year Strategy was developed by the NSW Marine Estate Management Authority to coordinate the management of the marine estate.

Will taking part in this study cost me anything, and will I be paid?

Other than your time, there are no costs associated with participating in this research, nor are there any payments.

What if I don’t want to take part in this study?

Participation in this research is entirely your choice. Only those people who give their informed consent will be included in the project. Whether or not you decide to participate is your decision and will not disadvantage you. There are no consequences for those who decide not to participate.

What if I participate and want to withdraw later?

We are not asking for any information that would personally identify you so it will not be possible for us to identify and remove your data once the survey has been submitted. Once your responses have been submitted, we will assume that you have consented to the use of your information.

What will happen to the information that I give you?

The outcomes of the research will be published in relevant reports. The outcomes may also be published in an appropriate scientific journal and/or presented at relevant conferences. NSW DPI may provide general information on their website/social media that relates to the project and its outcomes.

What should I do if I want to discuss this study further before I decide?

If you would like further information or if you are having technical difficulties please contact: Lynne Hayes E: lhayes@csu.edu.au P: 02 6933 2802

Who should I contact if I have concerns about the conduct of this study?

Charles Sturt University’s Human Research Ethics Committee has approved this project. If you have any complaints or reservations about the ethical conduct of this project, you may contact the Committee on (02) 6933 4213 or ethics@csu.edu.au. Any issues you raise will be treated in confidence and investigated fully, and you will be informed of the outcome.

What do I need to do next?

Please print this information sheet for your personal record and to refer to at any time.
Please read through the Consent Statements on the next page and if would like to continue, select yes to proceed to the questions.

Marine Biosecurity Awareness – Social research study

Consent

- I agree to participate in the above research project and give my consent freely.
- I understand that the project will be conducted as described in the Participant Information Sheet, a copy of which I have retained.
- I am over the age of 18.
- I understand that participation involves completing a questionnaire
- I understand I can withdraw from the project at any time and do not have to give any reason for withdrawing.
- I understand that my personal information will remain confidential to the researchers
- I have had the opportunity to have questions answered to my satisfaction.

By submitting your responses you are indicating consent to participate.

- ◯ Yes, I agree and would like to proceed to the questions
- ◯ No, I would not like to proceed to the questions.

Charles Sturt University GRAHAM CENTRE for Agricultural Innovation NSW GOVERNMENT Department of Primary Industries

Marine Biosecurity Awareness – Social research study

A bit about you and how you use your boat.

1. What gender do you identify with?

- ◯ Male
- ◯ Female
- ◯ Non-binary
- ◯ Rather not say

2. In which age bracket are you?

- ◯ 18-34
- ◯ 35-50
- ◯ 52-65
- ◯ 66-80
- ◯ Over 80

3. Can you please tell us the length of your boat?

- ○ Less than 5m (16ft)
- ○ 5m (16ft) to less than 10m (32ft)
- ○ 10m (32ft) to less than 20m (65ft)
- ○ 20m (65ft) to less than 30M (98ft)
- ○ 30m (98ft) or more

4. When in NSW, where is your boat usually moored? Please tell us :

The nearest town/suburb and; __________

The name of the waterway __________

5. How is your boat usually moored ? Please select all that apply.
At a:

- ☐ Marina
- ☐ Boating or yacht club
- ☐ Private Mooring
- ☐ Private Jetty
- ☐ Other (please specify)

____

6. Do you use your boat as a source of income?

- ○ Yes
- ○ No

6a. Please select which of the following describe/s how your boat is used as a source of income. Select all that apply.

- [ ] Hospitality cruising
- [ ] Sports Tourism
- [ ] Fishing charters
- [ ] Construction or maintenance
- [ ] Other (please specify)

7. What do you **MAINLY** use your boat for?

- ○ Cruising
- ○ Diving/snorkelling
- ○ Racing or sailing
- ○ Travel
- ○ Fishing
- ○ Live aboard
- ○ My work
- ○ Research
- ○ Other (please specify)

8. How long have you owned this boat for?

- ○ Less than 12 months
- ○ 1-5 years
- ○ More than 5 years

9. In **summer**, how often do you use your boat, both taking it out or spending time aboard?

- ○ Daily
- ○ At least once per week
- ○ At least once per month
- ○ Never

10. In **winter**, how often do you use your boat, both taking it out or spending time aboard?

- ○ Daily
- ○ At least once per week
- ○ At least once per month
- ○ Never

11. Where do you **MOSTLY** use your boat?

- ◯ Locally, mostly in the same waterway
- ◯ Large distances across NSW
- ◯ Interstate
- ◯ Internationally

11a. If you travel to other waterways with your boat, how many times a year would you do this?

11b. If travelling to other waterways, how far do you travel?

Charles Sturt University GRAHAM CENTRE for Agricultural Innovation NSW GOVERNMENT Department of Primary Industries

Marine Biosecurity Awareness – Social research study

What is your understanding of biofouling ?

12. In the past 24 months, how often have you:

	Never	Once	Twice	More than twice
Checked your boat for biofouling (organisms that attach to submerged parts of a vessel or equipment).	○	○	○	○
Had an antifouling paint coating applied or re-applied to the hull.	○	○	○	○
Slipped your boat for biofouling cleaning.	○	○	○	○
Had biofouling cleaned in the water.	○	○	○	○
Used a bilge water pump-out facility.	○	○	○	○
Checked/cleaned the equipment you use on your boat for biofouling, e.g. fishing equipment, anchors and chains, etc.	○	○	○	○
Removed biofouling yourself.	○	○	○	○

12a. If you responded ***once, twice or more than twice*** to any of the above, please answer the following question. If you responded never to all of the above, please skip the next question.

Where do you remove biofouling ?

- ☐ At a slipway or on land
- ☐ In the water by a dive company
- ☐ I have never done this

13. Have you ever reported the presence of unusual looking or suspected marine pest organisms in biofouling, or diseased or sick looking organisms in biofouling ?

- ○ Yes
- ○ No

If yes, who did you report to? What did you report?

14. When would you clean biofouling from your boat? Select all that apply.

- [ ] When the antifouling layer paint certificate has expired
- [ ] At a regular interval
- [ ] When biofouling organisms become visible on the boat
- [ ] Before leaving to go to another location
- [ ] Before returning home
- [ ] Other (please specify)
- [ ] When fuel running costs become too great
- [ ] When the boat needs other maintenance
- [ ] Before selling the boat
- [ ] Never

Charles Sturt University GRAHAM CENTRE for Agricultural Innovation NSW GOVERNMENT Department of Primary Industries

Marine Biosecurity Awareness – Social research study

14a. You selected that you would clean biofouling from your boat at a regular interval. Please specify the interval.

- More than once a year
- Once a year
- Once every two years
- Once every three years
- Once every 5 years
- More than once every 5 years?

15. Please indicate how serious you consider the following situations to be.

	Not at all serious	A little serious	Moderately serious	Very serious	Extremely serious
There was a disease outbreak or spread of marine pests in the river/ocean due to biofouling on YOUR boat	○	○	○	○	○
There was a disease outbreak or spread of marine pests in the river/ocean due to biofouling on OTHER PEOPLE'S boats	○	○	○	○	○

16. Please indicate how likely you consider the following situations are to occur.

	Not at all likely	A little likely	Moderately likely	Very likely	Extremely likely
Diseases and/or pests will spread in river/ocean water because of biofouling on YOUR boat	◯	◯	◯	◯	◯
Diseases and/or pests will spread in river/ocean water because of biofouling on OTHER PEOPLE'S boats	◯	◯	◯	◯	◯

17. Please select Yes or No for the following statements:

	Yes	No
I know that biofouling on boats can cause damage to the health of the waterways.	◯	◯
I know that I need to clean biofouling from my boat regularly.	◯	◯
I know that it is not just the hull but also other niche areas of my boat that can develop biofouling, such as engine cooling systems, bilge and ballast water tanks, the propeller and shaft and other water inlets and outlets.	◯	◯
I know that everyone has to report suspected marine pests or signs of aquatic diseases.	◯	◯
I know about the General Biosecurity Duty as part of NSW Biosecurity Act 2015.	◯	◯

18. In your own words, how would you define biosecurity?

19. Please use the scale to indicate your level of agreement with the following statements:

	Disagree	Neutral	Agree
I do not know how to carry out biofouling cleaning activities on my boat.	◯	◯	◯
I do not have the skill to carry out biofouling cleaning activities on my boat.	◯	◯	◯
I do not know where or who to contact regarding having biofouling cleaned from my boat.	◯	◯	◯
I did not know that I needed to carry out biofouling cleaning activities.	◯	◯	◯
I never remember at the right time to carry out/organise biofouling cleaning activities.	◯	◯	◯
I physically cannot carry out/organise biofouling cleaning activities on my boat.	◯	◯	◯

20. Please use the scale to indicate your level of agreement with the following statements:

	Disagree	Neutral	Agree
I do not have the time to carry out/organise biofouling cleaning activities on my boat.	◯	◯	◯
It is too expensive to conduct biofouling cleaning activities on my boat.	◯	◯	◯
I do not have access to shipyards and/or service providers to clean biofouling on my boat.	◯	◯	◯
Most of the people I know with moored boats do not clean biofouling from their boats.	◯	◯	◯
In my community, people who own moored boats are expected to clean biofouling from their boats or use service providers to get this done.	◯	◯	◯
I do not consider it necessary to clean biofouling from my boat.	◯	◯	◯
I am not sure if people I know with moored or berthed boats clean biofouling from them.	◯	◯	◯

21. Please use the scale to indicate your level of agreement with the following statements:

	Disagree	Neutral	Agree
I do not feel any reason to keep my boat clean of biofouling.	◯	◯	◯
Removing biofouling will not improve the running costs of my boat.	◯	◯	◯
Removing biofouling from my boat will not improve the health of the water ways.	◯	◯	◯

	Disagree	Neutral	Agree
I do not see any point to using an antifouling paint layer on my boat.	◯	◯	◯
I do not think that the threat from marine pest and biofouled boats to waterways are as bad as they are made out to be.	◯	◯	◯
I believe that biofouling cleaning techniques are ineffective in removing biosecurity risks from my boat.	◯	◯	◯
Having a clean boat is the right thing to do.	◯	◯	◯
Cleaning biofouling is a waste of time because the marine pests come back again.	◯	◯	◯
Removing biofouling from my boat and protecting the water ways are inseparable.	◯	◯	◯
Having a clean boat is part of me being recognised as a good boat owner.	◯	◯	◯
I would be less likely to go boating on others' vessels if I knew they had not managed the risk and cleaned their biofouling.	◯	◯	◯

22. If you had questions about an unusual marine animal/plant on your boat, who would you go to for information and advice? Select all that apply.

- [ ] DPI Biosecurity
- [ ] Local Fisheries office
- [ ] Roads and Maritime Services
- [ ] Local Land Services
- [ ] National Parks
- [ ] Port Authority of NSW
- [ ] Emergency Animal Disease hotline
- [ ] Other (please specify)

23. Have you ever received information and/or advice on pests and diseases posing a risk to the waterways?

- ○ Yes
- ○ No

Charles Sturt University | GRAHAM CENTRE for Agricultural Innovation | NSW GOVERNMENT Department of Primary Industries

Marine Biosecurity Awareness – Social research study

23a. For each of the sources of information and/or advice that you received, please indicate how useful it was. If you have not received information and advice, please select the N/A option (ie please don't leave any options blank)

	N/A	Not at all useful	A little bit useful	Somewhat useful	Very useful	Extremely useful
DPI Biosecurity	○	○	○	○	○	○
Local Fisheries office	○	○	○	○	○	○
Roads and Maritime Services	○	○	○	○	○	○
Local Land Services	○	○	○	○	○	○
National Parks	○	○	○	○	○	○
Port Authority of NSW	○	○	○	○	○	○
Emergency Animal Disease hotline	○	○	○	○	○	○

24. Please indicate your preferred method/s of information delivery about boat maintenance and waterway health. Select all that apply.

- [ ] One-on-one conversations
- [ ] Face-to-face workshops
- [ ] Print newsletters/fact sheets
- [ ] Electronic newsletters/fact sheets
- [ ] Smartphone applications (apps)
- [ ] Social media
- [ ] Websites
- [ ] Other (please specify)

25. How did you hear about this survey?

- [ ] Through a friend
- [ ] Webpage
- [ ] Facebook
- [ ] Other (please specify)
- [ ] Twitter
- [ ] LinkedIn
- [ ] Newsletter

26. If you have any comments related to the topics presented in this survey, please provide them here.

Thank you for your interest and input into our research. Please select the DONE button to end the survey.
